# A novel radiographic scoring system for growth abnormalities and structural change in children with juvenile idiopathic arthritis of the hip

**DOI:** 10.1007/s00247-018-4136-6

**Published:** 2018-05-01

**Authors:** Susan C. Shelmerdine, Pier Luigi Di Paolo, Jasper F. M. M. Rieter, Clara Malattia, Laura Tanturri de Horatio, Karen Rosendahl

**Affiliations:** 1 Department of Clinical Radiology, Great Ormond Street Hospital, London, WC1N 3JH UK; 2 UCL Great Ormond Street Institute of Child Health, London, UK; 3 Department of Radiology, Ospedale Pediatrico Bambino Gesu, Rome, Italy; 4 Department of Radiology, Academic Medical Center, University of Amsterdam, Amsterdam, the Netherlands; 5 Clinica Pediatrica Reumatologia, Istituto Giannina Gaslini, Genova, Italy; 6 Dipartimento di Neuroscienze, Riabilitazione, Oftalmologia, Genetica e Scienze Materno-Infantili, Università degli studi di Genova, Genova, Italy; 7 Department of Pediatric Radiology, Haukeland University Hospital, Bergen, Norway; 8 Department of Clinical Medicine, K1, University of Bergen, Bergen, Norway

**Keywords:** Children, Hip, Juvenile idiopathic arthritis, Measurements, Radiography, Repeatability, Severity scoring

## Abstract

**Background:**

Approximately 20–50% of children with juvenile idiopathic arthritis (JIA) have hip involvement within 6 years of diagnosis. Scoring systems for hip-related radiographic changes are lacking.

**Objective:**

To examine precision of potential radiographic variables and to suggest a scoring system.

**Materials and methods:**

We reviewed a set of 75 pelvic radiographs from 75 children with JIA hip involvement across two European centres. We assessed findings of (1) destructive change and (2) growth abnormality, according to a pre-defined scoring system. All radiographs were scored independently by two sets of radiologists. One set scored the radiographs a second time. We used kappa statistics to rate inter- and intra-observer variability.

**Results:**

Assessment of erosions of the femoral head, femoral neck and the acetabulum showed moderate to good agreement for the same reader (kappa of 0.5–0.8). The inter-reader agreement was, however, low (kappa of 0.1–0.3). There was moderate to high agreement for the assessment of femoral head flattening (kappa of 0.6–0.7 for the same reader, 0.3–0.7 between readers). Joint space narrowing showed moderate to high agreement both within and between observers (kappa of 0.4–0.8). Femoral neck length and width measurements, the centrum–collum–diaphysis angle, and trochanteric–femoral head lengths were relatively precise, with 95% limits of agreement within 10–15% of the observer average.

**Conclusion:**

Several radiographic variables of destructive and growth abnormalities in children with hip JIA have reasonable reproducibility. We suggest that future studies on clinical validity focus on assessing only reproducible radiographic variables.

## Introduction

Juvenile idiopathic arthritis (JIA) is an autoimmune disease of unknown origin, with an onset before the age of 16 years and a reported incidence of 1–2:1,000 children [1, 2]. It is characterised by synovial inflammation, with soft-tissue oedema and effusion of the involved joints, followed by destructive changes of cartilage and bone if treatment fails. Although early treatment with biologics and methotrexate does reduce the morbidity, reports addressing the long-term side effects are concerning [3, 4]. This highlights the need for accurate tools to monitor treatment response.

Approximately 20–50% of children with JIA, particularly those with systemic-onset disease, have hip involvement within 1–6 years of disease onset [5, 6]. Hip involvement is often a predictor of a severe disease course and carries a high risk of disability. Typically both hips are affected, but unilateral involvement sometimes occurs. Traditionally, conventional radiography has been used to detect, quantify and monitor osteochondral changes such as growth disturbances and destructive change (narrowed joint space from thinning and loss of cartilage, erosions, and avascular necrosis as a complication to treatment) [7–9]. Radiographic findings vary according to mode of onset and age; in younger children the initial findings might be developmental rather than destructive, i.e. overgrowth/flattening, with subsequent development of coxa magna and varus deformity, whereas in children with clinical hip involvement, destructive changes might supervene. Children with later-onset JIA sometimes have destruction/narrowed joint space as the first feature, often followed by development of protrusio acetabuli [5, 8, 10]. Premature closure with subsequent growth arrest might also occur [11].

Several scoring systems for radiographic JIA changes have been proposed; however most are based on hand and wrist radiographs [12–17]. Ideally a radiographic score for structural change should reflect the total burden of disease damage; however no generally accepted score, be it based on an “index” joint or a composite score, has been identified [12, 18].

Scoring systems for radiographic hip joint changes in children with JIA are lacking. One exception is a system by Bertamino et al. [18] in 2010, the Childhood Arthritis Radiographic Score of the Hip (CARSH), devised by a panel of five paediatric rheumatologists. Based on an evaluation of joint space narrowing, erosions, growth abnormalities, subchondral cysts, malalignment, sclerosis of the acetabulum and avascular necrosis of the femoral head, the hips were scored as 0 (normal) to 32 (extensive, destructive disease in both hips); based on high intra-class correlation, the authors concluded that the system was reliable [18]. However no information was given as to differences between or within scorers.

In the present study, our goal was to examine the repeatability of radiographic findings used in the CARSH scoring system: erosions, flattening of the femoral head, joint space, fovea assessment and sclerosis. Second, we aimed to validate additional markers for both growth abnormalities and structural damage, for the purpose of informing future scoring systems for structural hip damage.

## Materials and methods

This study is part of a large longitudinal multi-centre project (Health-e-Child) to establish imaging-based scoring systems for children with JIA with wrist or hip involvement. The project was approved by the institutional research ethics committees at Great Ormond Street Hospital, London, UK, and Ospedale Bambino Gesu, Rome, Italy, and written informed consent was obtained from all the patients or their caregivers.

For this particular study, we reviewed a subset of 75 hip radiographs from 75 children with JIA with hip involvement (59 seen at the outpatient clinic at Great Ormond Street Hospital during 2006–2016, and 16 seen at Bambino Gesu Hospital). This subset was chosen based on clinical demographic information to reflect a wide range of disease duration, JIA subset and severity in order to robustly test the variables within our radiographic scoring system.

All hip radiographs were scored by two sets of radiologists, blinded to each patient’s length of disease, prior imaging, clinical symptoms and subtype of JIA. The images were all scored once in consensus by an experienced paediatric radiologist (LTdH, 10 years of paediatric radiology experience) and general radiology consultant (PLdP, 5 years of paediatric radiology experience), and twice by consensus between an experienced paediatric radiologist (K.R., 25 years of paediatric radiology experience) and a paediatric radiology research fellow (S.C.S., 3 years of paediatric radiology experience). Two weekend calibration sessions were conducted prior to scoring, using imaging examples not included for analysis in this study.

### Scoring/measurements of radiographs

Based on a pelvic anteroposterior radiograph taken with the child supine and focused 2–3 cm above the pubic symphysis, all measurements and angles were assessed using a standard electronic measurement tool (for angles and distances) provided by the local picture archiving and communications software. The following features were scored for the right and left hips separately.

#### Destructive changes

**Fig. 1:**
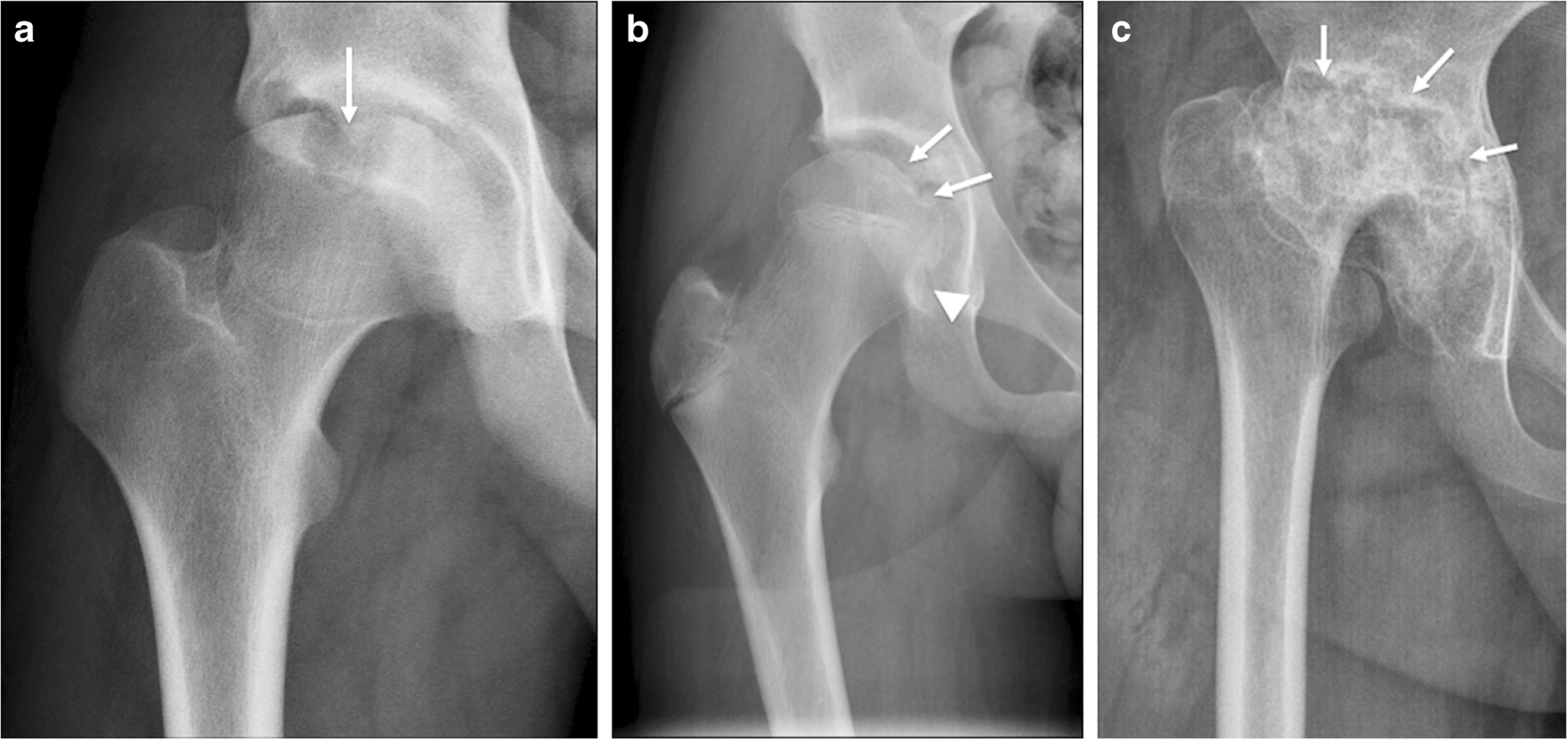
Measuring bone erosions through a variety of destructive changes seen at the hip joint on anteroposterior pelvic radiographs in different children with juvenile idiopathic arthritis. **a** Radiograph in a 16-year-old boy shows a large bone erosion at the weight-bearing portion of the proximal femoral epiphysis with associated sclerosis (*arrow*). **b** Radiograph in a 13-year-old boy shows a subtle bone erosion along the medial femoral neck, just inferior to the physis (*arrowhead*). Other small erosions are also present at the medial aspect of the proximal femoral epiphysis (*arrows*). **c** Radiograph in a 13-year-old girl shows multiple irregularities along both the acetabular roof and femoral head with loss of femoral head height (*arrows*), in keeping with widespread erosive changes

We measured bone erosions, flattening of the femoral head, enlargement of the fovea, presence of sclerosis and the height of the joint space. *Bone erosions* (Fig. 1) were defined as a bone depression with a cortical breach and these were evaluated at three locations: the femoral head (proportion of surface involved: 0=0%, 1=1–25%, 2=26–50%, 3=51–75%, 4=76–100%); the femoral neck (present or not); and the acetabulum (proportion of the acetabular surface involved: 0=0%, 1=1–33%, 2=34–66%, 3=67–100%).

**Fig. 2:**
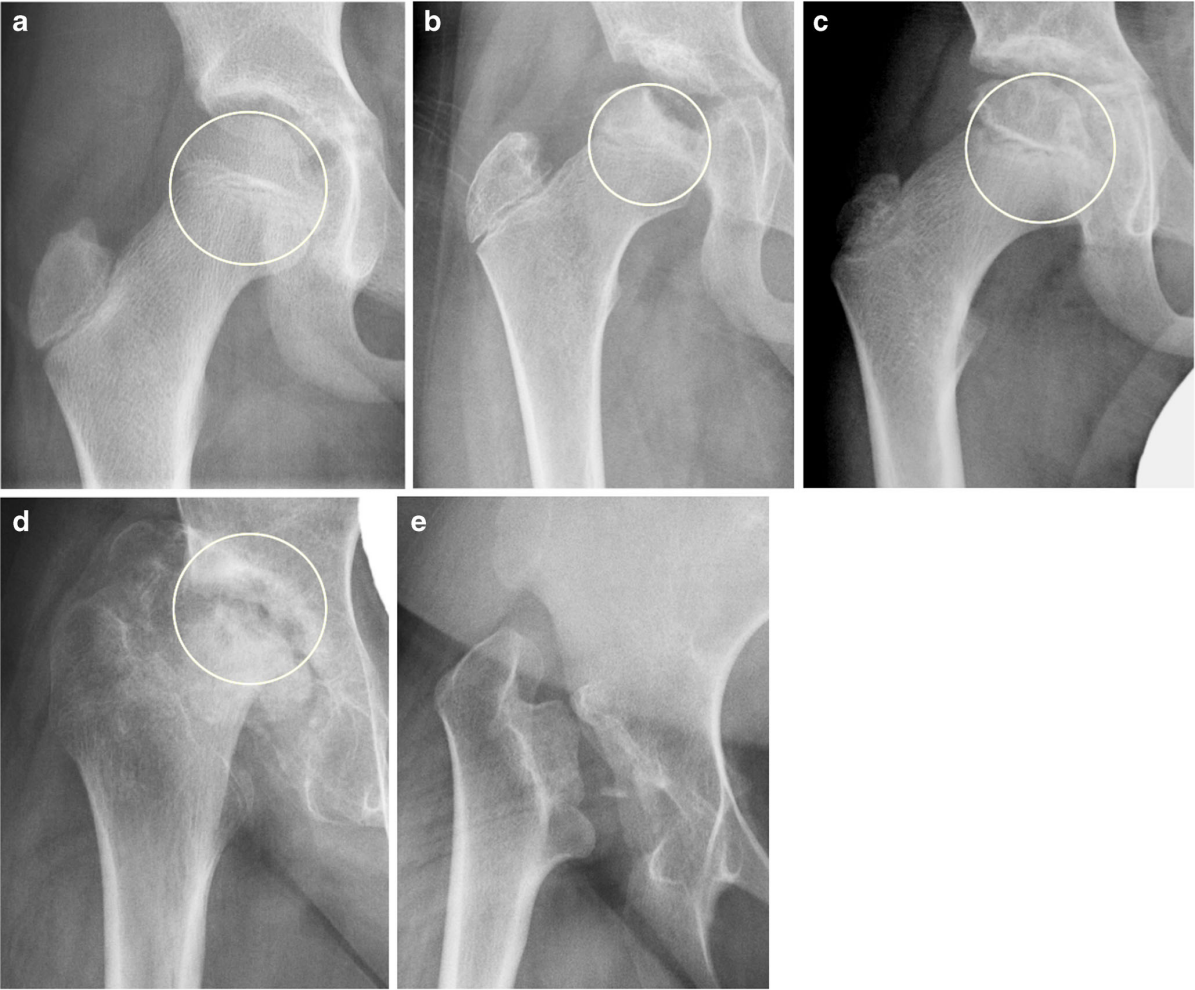
Femoral head flattening of differing severities in children and a young adult with juvenile idiopathic arthritis, as shown on an anteroposterior pelvic radiograph measured by the Mose template for reference. The Mose template in (**a–d**) is a circle drawn to represent where the femoral head should be located, and the degree of flattening is judged using a score of 0–4 according to 25% incremental losses in head height. **a** Radiograph in a 9-year-old boy shows normal femoral head without any loss of height (score = 0). **b** Radiograph in a 10-year-old boy shows mild loss of femoral head height of <25% (score 1). **c** Radiograph in a 9-year-old boy shows moderate loss of femoral head height of 26–50% (score 2). **d** Radiograph in a 13-year-old boy shows marked loss of femoral head height of 51–75% (score 3). **e** Radiograph in an 18-year-old woman shows total loss of femoral head height >75%. In this image the Mose template is not drawn because no residual femoral head is present (score 4)

*Flattening of the femoral head* (Fig. 2) was judged subjectively and by using a Mose template as a reference [19] (0–4, i.e. loss of height in increments of 25%). *Enlargement of the fovea* was scored as 0=normal, 1=potentially enlarged or 2=enlarged. *Presence of sclerosis* was rated at two locations — the femoral head and the acetabulum — as 0–4, i.e. in increments of 25%.

**Fig. 3:**
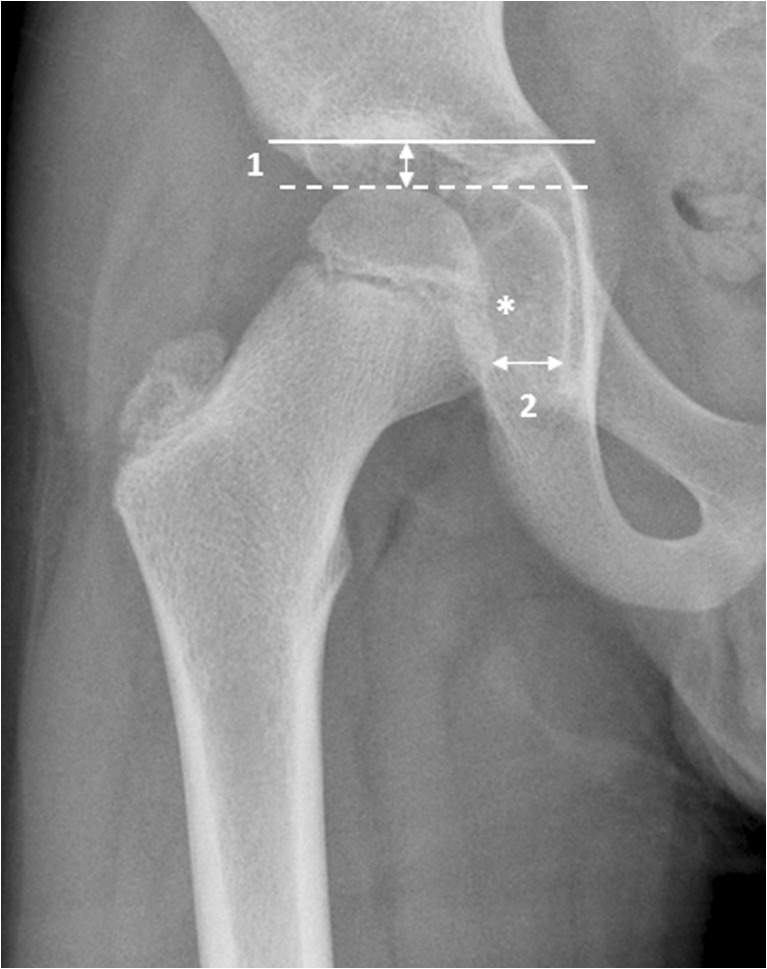
Demonstration of joint space measurements on an anteroposterior pelvic radiograph in a 9-year-old boy with juvenile idiopathic arthritis. We performed measurements in two locations: (1) superiorly, we drew a horizontal line along the roof of the acetabulum (*solid line*) and the most superior aspect of the femoral head (*dashed line*) and we calculated the distance between the two (*arrow labelled 1*); and (2) medially, we measured the joint space to the center of the femoral head (*arrow labelled 2*), just below the fovea (*)

**Fig. 4:**
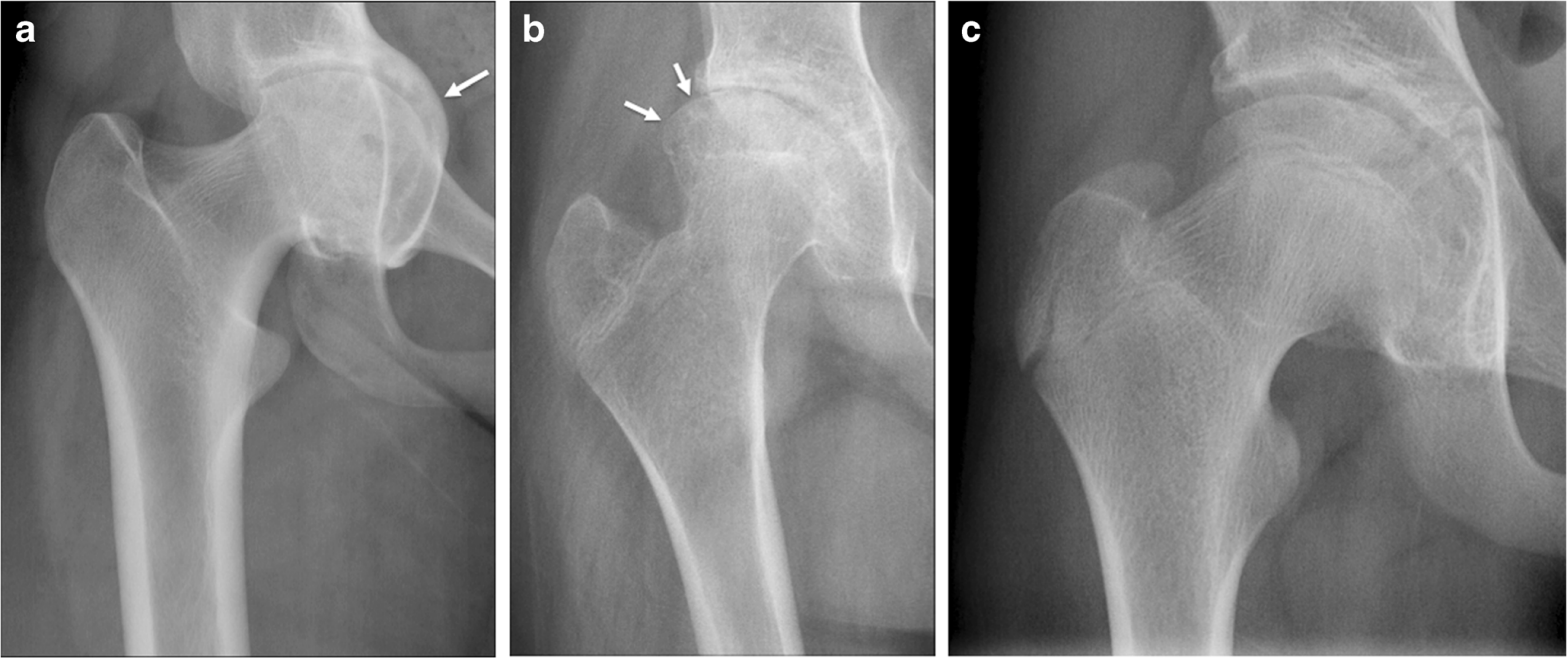
Abnormal bone remodeling at the hip joint as demonstrated on an anteroposterior pelvic radiograph in three adolescents with juvenile idiopathic arthritis. These changes were noted and commented upon during the scoring process as present or absent, although their severity was not allocated a score. **a** Radiograph in an 18-year-old woman shows protrusio acetabulae (*arrow*). **b** Radiograph in a 13-year-old girl shows lateral squaring of the superior femoral epiphysis, with associated loss of superior joint space (*arrows*). **c** Radiograph in a 14-year-old boy shows coxa magna of the femoral head, with a generally enlarged and widened superior capital epiphysis

*The height of the joint space* was measured at two locations (Fig. 3): cranially (mid-weight-bearing area) and medial to the center of the femoral head (just below the fovea). Moreover, the joint space height was categorised as normal (≥4 mm), mildly narrowed (2–4 mm) or narrowed (≤2 mm). The presence of ankyloses, subchondral cysts and protrusio acetabulae (Fig. 4) was noted (the medial aspect of the femoral head is medial to the ilio-ischial line [20]).

#### Growth abnormality

**Fig. 5:**
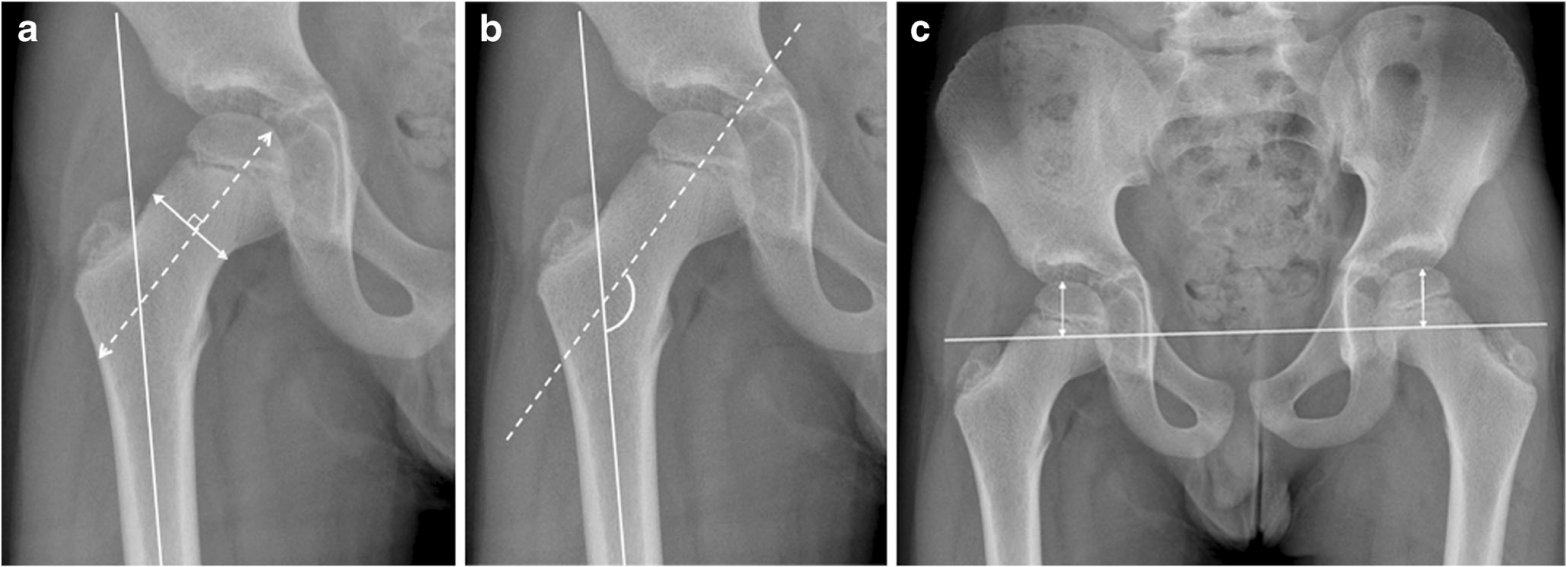
Proximal femoral measurements are demonstrated on anteroposterior pelvic radiographs in a 10-year-old boy with juvenile idiopathic arthritis **a** The femoral length (*dashed line*) was measured along the length of the femoral head, from the lateral aspect of the proximal femoral diaphyseal cortex to the femoral head. The femoral width (*solid line*) was drawn perpendicular to the dashed line for the femoral length, approximately midway along the femoral neck. **b** Centrum–collum–diaphysis angle represents the angle between the line drawn for the femoral length (*dashed line*), and a second line along the shaft of the femoral diaphysis (*solid line*). **c** The trochanteric femoral height is given as a separate measurement for the right and left femora. An intertrochanteric line (*horizontal line*) connecting both greater trochanters of the femora is drawn and a measurement from this line to the superior aspect of both the proximal femoral epiphyses is taken (*vertical lines*). This measurement should be perpendicular to the intertrochanteric line

We measured growth abnormality (Fig. 5) using the following criteria: (1) the length of the femoral neck, measured along the center of the femoral neck, from the trochanter area to the surface of the femoral head (in millimetres); (2) the width of the femoral neck (in millimetres; normal standards for joint space width by age are lacking); (3) varus/valgus deformity (projected head–neck–shaft [centrum–collum–diaphysis]) angle (in degrees), the trochanteric–femoral head height (in millimetres); and (4) the presence of closed physis, coxa magna or coxa brevis (yes/no).

The centrum–collum–diaphysis angle, measured from an anteroposterior radiograph, decreases with age, from approximately 145° in newborns to 135° at skeletal maturity. The term coxa vara is practically defined as a neck–shaft angle less than 120° [21] while coxa valga is diagnosed when the angle is increased, usually above 135°. These growth abnormality features were all measured to identify whether readers could reliably reproduce each variable. The readers were not asked to comment on whether the changes were abnormal or age-appropriate for the radiographs.

#### Normal variations

**Fig. 6:**
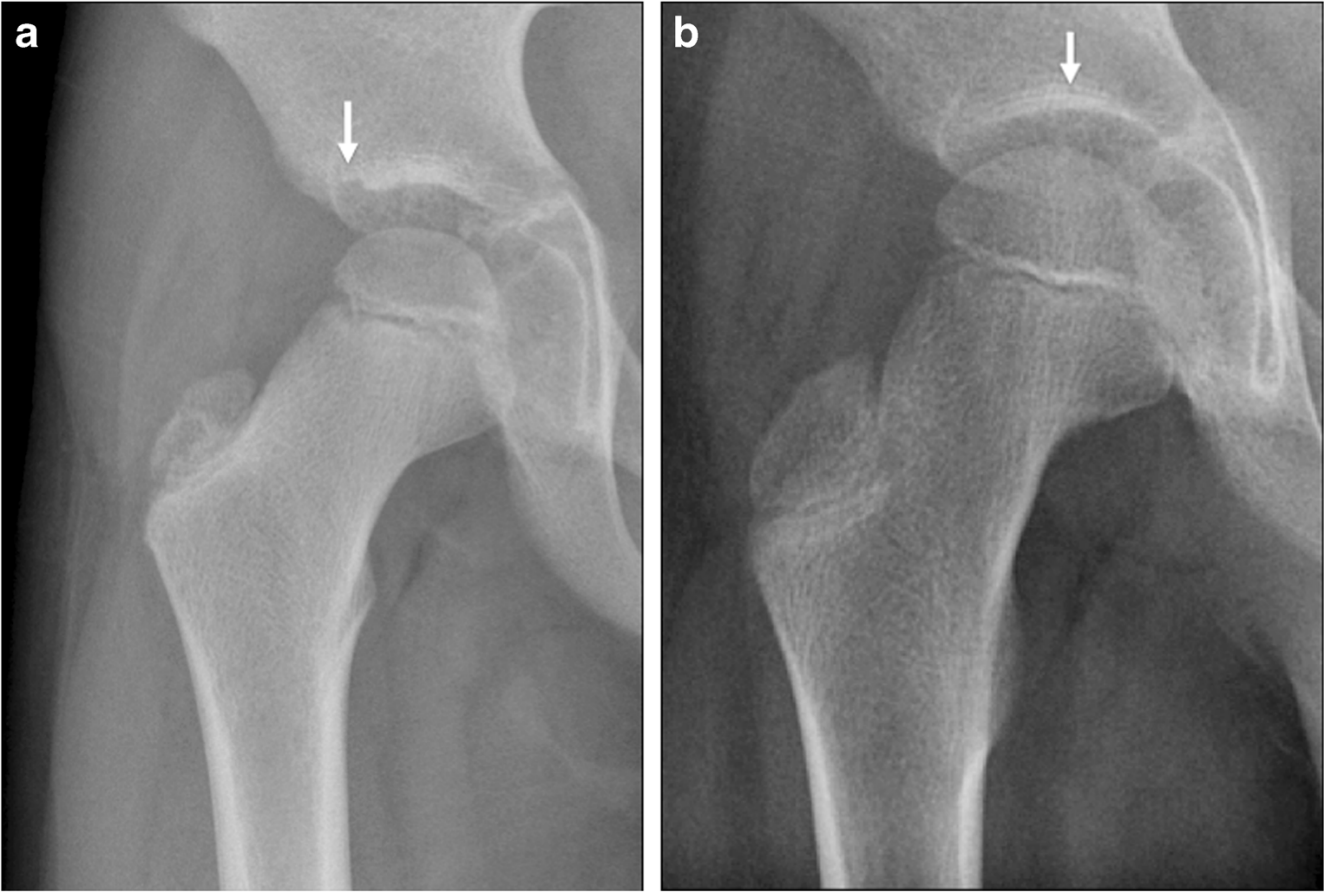
Normal variations of acetabular anatomy are demonstrated on an anteroposterior pelvic radiograph in two children with juvenile idiopathic arthritis. **a** Radiograph in a 10-year-old boy shows a small defect at the lateral aspect of the acetabular roof (*arrow*). **b** Radiograph in a different 10-year-old boy shows mild sclerosis at the lateral and weight-bearing areas of the acetabular roof (*arrow*)

The following features were considered to be within normal variation: lateral defect of the acetabulum**,** bone depression of the weight-bearing portion of the acetabular roof, and mild sclerosis of the weight-bearing area of the acetabulum (Fig. 6)**.**

### Statistical analysis

We analysed differences in scoring for each of the features separately, using the Cohen kappa statistics (simple kappa). A kappa of ˂0.2 was considered poor, 0.21–0.40 fair, 0.41–0.60 moderate, 0.61–0.80 good and 0.81–1.00 very good. We analysed differences in continuous variables using Bland–Altman plots and 95% limits of agreement. Statistical analyses were performed using predictive analytics software (SPSS version 23/24; IBM, Armonk, NY).

## Results

**Fig. 7:**
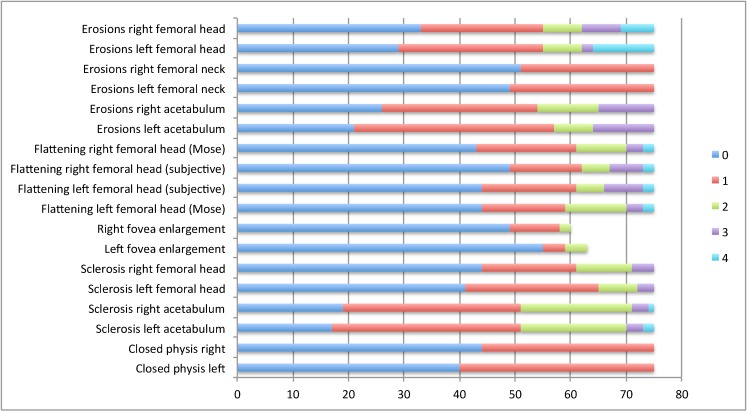
Overview of the radiographic scores for eight features assessed in 75 paediatric patients, right and left hips separately (based on first consensus reading). The x-axis denotes number of hips. The fovea was visible for comment in 63/75 left hips and 60/75 right hips

We included one set of radiographs from a total of 75 children (39 females), mean age 13 years 1 month (range 6–21 years) with hip JIA. Twenty-eight had the polyarticular form of JIA, while 23 had oligo JIA, 11 had enthesitis-related arthritis, 8 had systemic-onset JIA, 1 had psoriatic JIA and 4 had non-differentiated JIA. Mean duration of disease at the time the radiographs were obtained was 5.1 years (range 1 year to 15 years). The distribution of changes seen for right and left hips separately is shown in Fig. 7.

### Measures for destructive changes

Assessment of erosions of the femoral head, femoral neck and the acetabulum showed moderate agreement for the same reader, with kappa values of 0.5–0.8 except for the left acetabulum (Table 1). The inter-reader agreement was low, however, with kappa values of 0.1–0.3. There was a high to moderate agreement for the assessment of femoral head flattening, both subjectively and when using the Mose circle, with kappa values of 0.6–0.7 for the same reader and 0.3–0.7 between readers. The agreement for assessment of an enlarged fovea was poor, both within and between readers, with kappa values 0.1–0.4 (Table 1).

**Table 1:** Repeatability of features used for assessing destructive change and growth abnormalities radiographically in children and young adults with hip-related juvenile idiopathic arthritis^a^

**Markers of damage**	**Right hip**		**Left hip**	
	**Intra-reader kappa**	**Inter-reader kappa**	**Intra-reader kappa**	**Inter-reader kappa**
**Destructive change**				
Erosions of femoral head (0–4 scale)	0.6	0.3	0.6	0.2
Erosions of femoral neck (0–1 scale)	0.8	0.3	0.5	0.1
Erosions of acetabulum (0–3 scale)	0.5	0.1	0.3	0.2
Femoral head flattening (subjective, 0–4 scale)	0.7	0.4	0.7	0.3
Femoral head flattening (Mose circle, 0–4 scale)	0.6	0.4	0.6	0.7
Enlarged fovea (0–2 scale)	0.4	0.2	0.2	0.1
Sclerosis of acetabulum (0–4 scale)	0.6	0.1	0.6	0.2
Sclerosis of femoral head (0–4 scale)	0.3	0.5	0.6	0.4
Joint space narrowing (0–2 scale)	0.8	0.5	0.7	0.4
**Growth abnormality** ^**b**^				
Closed physis (no [0]/yes [1])	0.9	0.4	0.9	0.5

^a^Findings are given for categorical scoring features, using kappa statistics, based on 75 patients

^b^Based on the first 41 patients

The mean superior joint space height was 4.4 mm on the right side, with 95% limits of agreement of –1.4 mm to 3.8 mm and –4.9 mm to 2.3 mm within and between observers, respectively (Table 2). Categorising joint space width, on a 0–2 scale, into narrowed (<2 mm), possibly narrowed (2–4 mm) or normal (>4 mm) resulted in moderate to high agreement both within and between observers, with kappa values at 0.4–0.8 (Table 1). Sclerosis of the femoral head and subchondral cysts occurred in very few cases, thus we could not accurately assess repeatability.

**Table 2:** Repeatability of measured features used for assessing destructive change and growth abnormalities radiographically, in patients with hip-related juvenile idiopathic arthritis^a^

**Markers of damage**	**Right hip**			**Left hip**		
	**Mean**	**95% LOA Intra-reader**	**95% LOA Inter-reader**	**Mean**	**95% LOA Intra-reader**	**95% LOA Inter-reader**
**Destructive change**						
Joint space, superior aspect, mm	4.4	-1.4–3.8	-4.9–2.3	5.1	-1.4–1.4	-0.7–6.1
Joint space, medial aspect, mm	7.3	-4.8–5.4	-4.5–4.9	6.9	-3.8–3.7	-4.8–4.5
**Growth abnormality** ^**b**^						
Femoral neck length, cm	7.7	-1.1–0.5		7.6	-1.4–0.6	
Femoral neck width, cm	3.3	-0.2–0.2		3.5	0.2–0.3	
Projected centrum–collum–diaphysis angle, degrees	135.2	-11.4–3.1		135.2	-10.3–2.9	
Trochanteric femoral head length, cm	2.3	-0.2–0.2		2.3	-0.2–0.2	

*cm* centimetres, *LOA* limits of agreement, *mm* millimetres

^a^Based on 75 patients

^b^Based on the 41 first cases

### Measures for growth abnormalities

The mean measures for the femoral length and width, the centrum–collum–diaphysis angle and trochanteric–femoral head length are given in Table 2. Their 95% limits of agreement lay within 10–15% of the scorer average (Table 2). Coxa magna, coxa brevis and protrusio acetabuli were seen in 12, 1 and 11 hips, respectively, thus we could not assess repeatability. None of the hips was subluxated or dislocated.

## Discussion

We have identified a set of radiographic markers suggestive of chronic disease in children with hip JIA. The markers include the assessment of destruction, such as erosions of the femoral head and neck, flattening of the femoral head and joint space narrowing, as well as measures of growth abnormalities such as length and width of the femoral neck, projected centrum–collum–diaphysis angle and trochanteric–femoral head length. Their clinical validity remains to be determined.

Although subjective assessment of femoral head flattening performed well for the same observer, using Mose circle seemed to be a more robust method between assessors. This technique has been validated for use in other paediatric hip diseases such as Perthes disease and avascular necrosis [22]. The agreement for scoring erosions of the femoral head and neck using 0–4 scale and 0–1 scales, respectively, was moderate to good within readers, while the agreement for acetabular erosions performed poorer. Agreement between observers was only fair, underscoring the importance of reader calibration workshops and standardising radiographs when performing clinical trials.

Compared to the other radiographic hip scoring system in the literature for JIA in children (the CARSH score), of the seven features that were included [18] — joint space narrowing, erosions, growth abnormalities, subchondral cysts, malalignment, sclerosis of the acetabulum and avascular necrosis of the femoral head — we found moderate agreement for assessment of erosions and moderate to good agreement for assessment of joint space narrowing on a 0–2 scale, while scoring of acetabular sclerosis performed rather poorly. On the other hand, direct measurement of joint space height turned out to be rather inaccurate, with significant variation for the same observer. The variation was even higher for the medial joint space width, reflecting difficulties in identifying precise measurement points. Although we were aware of the large specter of normal, acetabular appearances, such as an arched roof, a flat roof or an angular roof, the different shapes might have biased the measurements. Whether joint space varies according to gender, age or weight has been addressed in a few studies, with no conclusions [23]. Although reference ranges by age are lacking, joint space narrowing is still considered an important radiographic marker of destructive disease in an arthritic joint [24].

Given our results, the high inter-observer agreement reported for the CARSH score, with intra-class correlation of 0.98 for baseline scores, 0.76 for changes in scores from baseline to time 1, and 0.96 for scores obtained for the whole dataset, are surprising [18]. These results might, however, be flawed because the assumption of independent measurements was violated, and, further, the hip radiographs were analysed chronologically by readers not blinded for previous findings/scores. Moreover, the dataset did not include normal cases. The joint space narrowing, believed to be the most characteristic radiographic finding in JIA, was assessed subjectively by comparing the two hips for each of the patients. If both sides were judged to be involved, consensus was made by pediatric radiologists. In sum, all these factors might have influenced the results.

Further, in the CARSH study the authors performed a comparison between clinical and radiographic disease markers [18]. Radiographic changes from baseline to the first follow-up were moderately correlated to clinical disease markers at the final follow-up, and to the radiographic long-term outcome. Interestingly, there was a poor association between the CARSH-score and clinical disease markers such as the Childhood Health Assessment Questionnaire (CHAQ) and index wrist radiographic assessments using Sharp van der Heijde and Poznanski scores [18]. The lack of correlation with wrist radiographic scores suggests that damage scores in the hand and wrist joints do not reliably reflect damage of the hip, underscoring the need for a hip-specific radiographic score.

Our findings compare well with what has been shown for the immature wrist joint, in that the appearances of chronic change differ from those in adult rheumatoid arthritis. In rheumatoid arthritis, chronic change manifests with bone erosions, while in immature bones, increased growth might succeed bony angulation and squaring, followed by carpal crowding and bone loss — with or without erosions. The different routes of bone destruction are caused by the large amount of cartilage still existent in immature bones.

In our image analysis, we included markers of growth abnormality to reflect the chronic changes specific to a paediatric population. The clinical validity of these features must still be assessed, particularly the centrum–collum–diaphysis angle given that it relies on patient positioning (e.g., an external rotation of the leg or marked antetorsion of the femur projects the angle incorrectly). It is also known that there is a high variance reported in the medical literature for measuring this angle, with inconsistency in how radiologists and orthopaedic surgeons account for this [25]. In addition, it is also doubtful whether coxa valga is a true, primary feature of JIA because it is more likely secondary to prolonged immobilisation from disuse secondary to non-weight-bearing [26].

Our study also had several limitations. First, there is the subjective nature of any radiographic scoring and measurements, with inherent biases in readers’ experiences and understanding of the factors required to score. We endeavoured to overcome this by hosting a calibration session among all readers prior to scoring and analysis, and conducting our scoring using consensus between two groups of two readers, although we understand that consensus reads with two readers might be less representative of clinical practice. Despite our efforts, challenges still exist in measurement of joint spaces by radiograph in skeletally immature patients whose epiphyseal cartilage is not well distinguished and also in potential differences in calibration and magnification when using electronic measurement tools on differing image viewing systems.

Our study also does not address the clinical validity of our scoring variables; however this was not our intention. Our intention was primarily to understand which imaging factors radiologists agree upon as robust markers in JIA with the desire to then assess these for clinical validity, based on the understanding that these features could be more reliably scored. The strengths of this study include the use of cases from two European centres and the multireader aspect of our data analysis. We include a breadth of pathology and severity of imaging features, which were assessed by a scoring system that tested a wide variety of radiographic features of JIA.

Final important considerations for further work in providing a robust radiographic hip-JIA scoring system, particularly pertaining to clinical validity, could include the discovery that differing radiographic features might not be equally representative of significant long-term damage or clinical deterioration, and that perhaps only specific reproducible measurements are the key to prognosticating outcomes on paediatric radiographs rather than a score. Nevertheless, we are unable to define these without further assessment and long-term data, and in this paper we demonstrate specific markers that can be assessed with some degree of reproducibility to take forward. The combination and utilisation of combined imaging features from differing modalities (e.g., ultrasound, MRI and osteodenistometry) might provide an opportunity to refine and stratify clinical outcomes; however this has not been established and is beyond the remit of this study. We hope that this information can be incorporated into future scoring systems to test clinical validity and applicability to other patient populations.

## Conclusion

Despite efforts at robust standardisation of measurements for different paediatric hip radiograph variables associated with JIA, there remains poor inter-observer variability, although reasonable intra-observer variability. We have identified several features that are more reliable for reproducible measurements and hope that these features can be helpful for use in studies that assess clinical validity and long-term patient outcomes.
